# Untreated PKU Patients without Intellectual Disability: What Do They Teach Us?

**DOI:** 10.3390/nu11112572

**Published:** 2019-10-25

**Authors:** Danique van Vliet, Annemiek M.J. van Wegberg, Kirsten Ahring, Miroslaw Bik-Multanowski, Kari Casas, Bozena Didycz, Maja Djordjevic, Jozef L. Hertecant, Vincenzo Leuzzi, Per Mathisen, Francesca Nardecchia, Kimberly K. Powell, Frank Rutsch, Maja Stojiljkovic, Fritz K. Trefz, Natalia Usurelu, Callum Wilson, Clara D. van Karnebeek, William B. Hanley, Francjan J. van Spronsen

**Affiliations:** 1 University of Groningen, University Medical Center Groningen, Beatrix Children’s Hospital, 9700 RB Groningen, The Netherlands; d01.vliet@umcg.nl (D.v.V.);; 2 Department of Gastroenterology, Radboud University Medical Center, 6500 HB Nijmegen, The Netherlands; 3 Department of PKU, Kennedy Center, Copenhagen University Hospital, DK-2600 Glostrup, Denmark; kirstenahring@hotmail.com; 4 University Children’s Hospital, Jagiellonian University, 30-663 Krakow, Poland; miroslaw.bik-multanowski@uj.edu.pl (M.B.-M.); bozenadidycz@wp.pl (B.D.); 5 Medical Genetics, Sanford Health, Fargo, ND 58102, USA; Kari.Casas@SanfordHealth.org; 6 Mother and Child Health Care Institute of Serbia Dr Vukan Cupic, School of Medicine, University of Belgrade, 11070 Belgrade, Serbia; mayamarko@gmail.com; 7 Department of Pediatrics, Tawam Hospital, 15258 Al-Ain, UAE; jhertecant@seha.ae; 8 Department of Pediatrics, Child Neurology and Psychiatry, Sapienza University of Rome, 00185 Rome, Italy; vincenzo.leuzzi@uniroma1.it (V.L.); francesca.nardecchia@uniroma1.it (F.N.); 9 Department of Internal Medicine, Oslo University Hospital, 0318 Oslo, Norway; pmathise@ous-hf.no; 10 Department of Genetics and Metabolism, Chapel Hill hospital, University of North Carolina, Chapel Hill, NC 27599-7487, USA; kpowel34@NCCU.EDU; 11 Department of General Pediatrics, Muenster University Children’s Hospital, 48149 Muenster, Germany; frank.rutsch@ukmuenster.de; 12 Institute of Molecular Genetics and Genetic Engineering, University of Belgrade, 11000 Belgrade, Serbia; maja.stojiljkovic@yahoo.com; 13 Dietmar-Hopp Metabolic Center, University Children’s Hospital, 69120 Heidelberg, Germany; friedrich.trefz@gmx.de; 14 Institute of Mother and Child, Centre of Reproductive Health and Medical Genetics, MD-2060 Chisinau, Moldova; natalia.usurelu@yahoo.com; 15 Newborn Metabolic Screening Unit, LabPlus, Auckland City Hospital, Auckland 1142, New Zealand; CallumW@adhb.govt.nz; 16 Departments of Pediatrics and Clinical Genetics, Emma Children’s Hospital, Academic Medical Centre, 1105 AZ Amsterdam, The Netherlands; c.d.vankarnebeek@amc.uva.nl; 17 Department of Pediatrics, Centre for Molecular Medicine, and Department of Pediatrics, Radboud University Medical Centre, 6525 XZ Nijmegen, The Netherlands; 18 Therapeutics, University of British Columbia, Vancouver, BC V5Z 4H4, Canada; 19 Clinical and Biochemical Genetics, Department of Pediatrics, The Hospital for Sick Children and the University of Toronto, Toronto, ON M5G 1X8, Canada; whanley@pathcom.com

**Keywords:** phenylketonuria, late-treated, untreated, outcome, brain vulnerability, inter-individual differences

## Abstract

Phenylketonuria (PKU) management is aimed at preventing neurocognitive and psychosocial dysfunction by keeping plasma phenylalanine concentrations within the recommended target range. It can be questioned, however, whether universal plasma phenylalanine target levels would result in optimal neurocognitive outcomes for all patients, as similar plasma phenylalanine concentrations do not seem to have the same consequences to the brain for each PKU individual. To better understand the inter-individual differences in brain vulnerability to high plasma phenylalanine concentrations, we aimed to identify untreated and/or late-diagnosed PKU patients with near-normal outcome, despite high plasma phenylalanine concentrations, who are still alive. In total, we identified 16 such cases. While intellectual functioning in these patients was relatively unaffected, they often did present other neurological, psychological, and behavioral problems. Thereby, these “unusual” PKU patients show that the classical symptomatology of untreated or late-treated PKU may have to be rewritten. Moreover, these cases show that a lack of intellectual dysfunction despite high plasma phenylalanine concentrations does not necessarily imply that these high phenylalanine concentrations have not been toxic to the brain. Also, these cases may suggest that different mechanisms are involved in PKU pathophysiology, of which the relative importance seems to differ between patients and possibly also with increasing age. Further research should aim to better distinguish PKU patients with respect to their cerebral effects to high plasma phenylalanine concentrations.

## 1. Introduction

Phenylketonuria (PKU; OMIM 261600) management is aimed at reducing plasma phenylalanine (Phe) concentrations as timely as possible and further keeping them within the recommended target range [1,2,3]. None of these guidelines take personalized medicine as a starting point, whereas it can be questioned whether universal plasma Phe target levels would result in optimal neurocognitive outcomes for all individual PKU patients. Similar plasma Phe concentrations do not seem to have the same consequences to the brain for each PKU individual. While the usual picture of untreated PKU includes developmental delay in development, resulting in severe intellectual disability and behavioral problems, this does not seem to be the case for all PKU patients. The most distinctive examples of this are the (often old) reports of untreated or late-diagnosed PKU patients, who have somehow escaped from intellectual disability despite high plasma Phe concentrations [4]. The mechanism underlying these differences in intellectual outcome between PKU patients still remains to be elucidated, as well as its implications for the optimal treatment of PKU individuals.

To better understand the inter-individual differences in brain vulnerability to high plasma Phe concentrations, PKU patients with a phenotype of a low vulnerability to high plasma Phe concentrations with respect to neurocognition would be informative and worth investigating in further detail. Therefore, as a first step, we aimed to identify such “unusual” PKU patients who are still alive, being untreated and/or late-diagnosed PKU patients with (near-)normal neurocognitive outcome despite high plasma Phe concentrations, to investigate their outcome with regard to other brain functions. Here, we describe 16 cases of such “unusual” PKU patients.

## 2. Cases

To identify PKU patients with unexpected favorable outcomes despite being untreated or late-diagnosed with high plasma Phe concentrations, who had not been described previously, we contacted physicians and dietitians personally, through a request on the metab-l server, and by presenting the idea for this study at the SSIEM (Society for the Study of Inborn Errors of Metabolism) and ESPKU (European Society for Phenylketonuria and Allied Disorders Treated as Phenylketonuria) in 2016. In total, we identified 16 cases of late-diagnosed PKU patients without intellectual disability despite high plasma Phe concentrations (>1200 µmol/L). Identified cases were diagnosed with PKU either following the diagnosis in an affected sibling (*n* = 4), because of a child of the mother being diagnosed with PKU by newborn screening or the maternal PKU syndrome (*n* = 6), or because of mental or neurological deterioration at a later age (*n* = 4). The most illustrative cases are presented here (Table 1). Other cases are described as Supplemental Material.

### 2.1. Detected through Affected Sibling

Cases 1A and 1B were born as the daughter (1A) and son (1B) of two non-consanguineous Dutch parents before the introduction of newborn screening. The boy presented at the age of 2 years and 1 month with developmental delay. His plasma Phe concentration was 1700 µmol/L and his electroencephalography (EEG) showed diffuse changes in cerebral functioning with epileptic features, without a clinical history of seizures. Dietary treatment was promptly initiated, and a neuropsychological examination 4 months later showed a developmental quotient (DQ) of 33. Genetic testing showed compound heterozygosity for the *PAH* variants c.829T > G/p.Y277D and c.1315+1G > A/p.IVS12+1G > A. Some years later, his elder sister, 1A, was also investigated and unexpectedly found at the age of 8 years and 10 months to have PKU with a plasma Phe concentration of 1800 µmol/L. The neurological examination showed no abnormalities. At school, she was noted to be slow and have difficulties focusing, but otherwise she was a normally developing child. Formal evaluation 2 months after the initiation of dietary treatment revealed neurocognitive functioning within the normal range. Subsequent evaluations at age 13 years and 49 years showed an IQ of 102 and 105, respectively, while treatment has always been problematic and was completely abandoned from 35 years of age. In contrast, neuropsychological evaluations of 1B at age 5 years and 9 months, 6 years and 6 months, 12 years, 40 years, and 47 years showed DQs/IQs of 72, 87, 99, 79, and 105, respectively, as assessed by different neuropsychological instruments.

Case 2 is a female originating from the USA who was unexpectedly found to have a blood Phe concentration of 2400 µmol/L at 9 years of age, after her younger brother had been diagnosed with PKU as part of an evaluation for severe intellectual disability at 3 years of age. She had no history of seizures and showed a (low-)normal IQ. No dietary treatment was applied. In contrast to her brother, who was institutionalized, she finished high school. However, she was unable to hold jobs due to behavioral issues. At 44 years of age, she presented with intermittent headaches, astigmatism, poor focus, anger outbursts, anxiety, depression, panic attacks, and episodes of screaming and disorientation at night. The blood Phe concentration at that time was 1651 µmol/L, which effectively decreased following the institution of dietary treatment.

Cases 3A and 3B were born as the children of healthy Polish parents. The eldest (3A) is a girl who first showed some mildly delayed psychomotor development, but later obtained very good results at school. At 10 years of age, she was unexpectedly found to have a plasma Phe concentration of >1200 µmol/L, after her younger brother (3B) had been diagnosed with PKU. His psychomotor development had been retarded: he sat alone at 12 months, walked at 17 months, and said his first words at 4 years of age. In addition, he showed hyperactivity and excessive mobility. At 4.5 years of age, he was investigated by the neurologist because of speech delay, which revealed a plasma Phe concentration of 2100 µmol/L. Following diagnosis in both children, dietary treatment was instituted in both patients. The older sister obtained a linguistic higher-education degree, and the younger brother obtained very good results at school, as well. Genetic analyses showed a compound heterozygosity for the *PAH* variants c.1222C > T/p.R408W and c.782G > A/p.R261Q.

### 2.2. Mothers Detected through Their Child Suffering from PKU or the Maternal PKU Syndrome

Case 4 is a woman of (low-)normal intelligence from New Zealand who had completed high school and has worked as an enrolled nurse. She presented at the age of 27 years after she had given birth to a child with features of the maternal PKU syndrome. She was diagnosed with PKU with a plasma Phe concentration of 1500 µmol/L, which was confirmed by a genetic analysis that showed a compound heterozygosity for the c.311C > A/p.A104D and c.1315+1G > A/p.L249F *PAH* variants. Treatment was not started until the age of 46 years. On dietary treatment, with plasma Phe concentrations <400 µmol/L, she started to show some deterioration in function. Therefore, treatment was discontinued at 49 years of age, but she continued to deteriorate during her 50s and early 60s, showing moderate/severe intellectual disability, Parkinson’s features, rigidity, tremors, and depression. Cerebrospinal fluid (CSF) analysis showed a Phe concentration of 490 µmol/L (reference values: 2–19 µmol/L), a tyrosine concentration of 22 µmol/L (reference values: 2–14 µmol/l), and a glutamine concentration of 776 µmol/L (reference values: 284–566 µmol/L) at plasma concentrations of respectively 1521, 64, and 502 µmol/L. Other amino acid concentrations in CSF were normal. Magnetic resonance imaging (MRI) showed mild generalized cerebral atrophy and moderate T2 white matter hyperintensities.

Case 5 is a Moldovan woman who completed general school till high school (9 classes) and is employed. She has a history of frequent headaches, intracranial hypertensive syndrome, some learning troubles at school, and irritative changes on EEG, with residual encephalopathy without an effect of anticonvulsive treatment. She presented at 23 years of age, after she had given birth to two children who had died because of congenital heart malformations. Because of her gynecological history, she was investigated for hyperhomocystinemia, which did not only identify elevated homocysteine concentrations, but also a plasma Phe concentration of 1293 µmol/L. The diagnosis of PKU was confirmed by compound heterozygosity for the *PAH* variants c.1222C > T/p.R408W and c.143T > C/p.L48S. At the moment of diagnosis, she was 4–5 weeks into her third pregnancy, and she decided to continue the pregnancy. Dietary treatment was initiated at 6–7 weeks of gestation, resulting in plasma Phe concentrations within the recommended range from 7–8 weeks of pregnancy onward. Her child was born with a very minor aortic coarctation, but suddenly died because of pneumonia. Brain imaging by computed tomography (CT) at 25 years of age showed no abnormalities, while neurocognitive assessment at 26 years showed an IQ of 81. Neuropsychological/behavioral assessment showed her to be distrustful of the motives of others and to have poorly controlled emotions and increased verbal aggression (having a grudge and worrying about the reason her children had died). Neurological examination revealed polyneuropathy.

### 2.3. Mental/Neurological Deterioration at Later Age

Case 6 is an Italian female and high school graduate, who presented at the age of 26 years with worsening headaches, scintillating scotoma, photophobia, and phonophobia, without a history of seizures. As part of the diagnostic workup, she was found to have PKU with a plasma Phe concentration of 2580 µmol/L and a compound heterozygosity for the *PAH* variants c.143T > C/p.L48S and c.781C > T/p.R261*. Following the introduction of the Phe-restricted diet, cephalgic episodes disappeared. Brain MRI showed leukoencephalopathy with bilateral white matter hyperintensities on delayed phase (DP), T2, and fluid-attenuated inversion recovery (FLAIR) in ventricular fronto-temporo-parietal and semioval centers. Neurological examination at 34 years of age did not show any abnormalities, and she has no behavioral or psychiatric symptoms.

Case 7 is a daughter of a Greek father and Norwegian mother, who was healthy during childhood and became an assistant nurse. She has given birth to a daughter who suffers from epilepsy and a moderate cognitive defect, as well as two additional children who exhibited microcephaly and a heart anomaly and died as newborns. From the age of 41 years, she suffered from headaches, depression, anxiety, tiredness, and lack of energy, and she experienced increasing difficulty performing practical tasks. She also had short episodes with confusion/loss of memory and involuntary contractions in facial muscles. As part of a comprehensive neurological examination at the age of 52 years, she showed a plasma Phe concentration of 1700 µmol/L. The diagnosis of PKU was further confirmed by compound heterozygosity for the *PAH* variants c.194T > C/p.I65T and c.311C > A/p.A104D. Neuropsychological examination revealed mild cognitive difficulties, mainly affecting attention, executive function, and other nonverbal capacities, consistent with diffuse cerebral affection as seen in untreated PKU. The brain MRI showed extensive white matter changes with multiple micro hemorrhages, and an EEG showed possible epileptogenic activity. On dietary treatment, she exhibited a gradual clinical improvement, becoming more energetic and less tired, and having less headaches. After one year of dietary treatment, there was a partial improvement of the white matter lesions and no epileptogenic activity on EEG. Repeated neurological examinations, however, showed regression of pathological findings on the brain MRI and EEG.

Case 8 is an Iranian male who moved to Norway at 10 years of age. He had a history without seizures, but suffered from dyslexia and social phobia form an early age. He has a bachelor degree in social science, completed studies in Farsi, and published a collection of poems. From adolescence, he showed tremor and slight gait problems, and at the age of 29 years, he was found to have a plasma Phe concentration of 1400 µmol/L. The diagnosis of PKU was further confirmed by compound heterozygosity for the *PAH* variants c.168G > T/p.E56D and c.782G > A/p.R261Q. A neuropsychological examination revealed nonverbal learning difficulties and dyslexia, slow processing speed performance, and strong verbal and reasoning abilities, with an IQ score of 82 (WAIS-IV). His brain MRI showed extensive signal changes in white matter, and his EEG showed slightly pathologic frontotemporal peak potentials, without epileptogenic activity. After initiation of dietary treatment, his attention and executive functioning slightly improved, but his tremor and gait problems remained unchanged.

## 3. Discussion

This study describes 16 cases of PKU patients without ID, despite a late diagnosis with high plasma Phe concentrations. Most notable is the fact that we could identify so many cases with favorable outcomes that had remained unreported and in which the patients are still alive. The second important observation was that, although these patients had intellectual functioning within the normal range, many showed other cerebral PKU symptoms, with a large heterogeneity with respect to severity and type of cerebral symptoms. The third remarkable finding was that, in some of the PKU patients, neurological symptoms only started in adulthood.

The textbook story of PKU states that untreated PKU results in severe intellectual disability, seizures, and psychiatric symptoms at an early age. Thanks to the introduction of newborn screening and dietary treatment, severe intellectual disability can be prevented [5]. In consequence, the focus of research has shifted toward further optimizing treatment. The question arises, however, whether further optimizing treatment should not also take into account the “original” natural history of PKU, acknowledging the large heterogeneity with respect to both the severity and type of cerebral symptoms.

In 1960, Knox reported that, in a series of *n* = 466 untreated PKU patients, 64.4% had an IQ < 20 and 23.2% had an IQ between 21 and 40, while only 12.4% had an IQ > 40 (9.7% had IQs of 41–60; 1.9% had IQs of 61–80; and 0.6% had IQs > 80) [6]. By this, Knox did not only show the severity of IQ impairment, but also the heterogeneity of the effect of PKU on intellectual functioning. By reviewing the literature, we recently identified 59 “unusual” reported cases of late-diagnosed PKU patients without severe intellectual disability despite high plasma Phe concentrations [4], clearly demonstrating that the severity of brain dysfunction is different for individual PKU patients. As reported here, we were able to identify 16 additional (and previously unreported) cases of “unusual” PKU patients who are still alive. With the introduction of newborn screening in the 1960s and 1970 in most developed countries, one might say that these phenotypical differences between untreated PKU patients have become irrelevant. However, the biological mechanisms underlying these differences in brain vulnerability to high plasma Phe concentrations should still be present in today’s population of early treated PKU patients, though being even more difficult to identify. If we aim to further optimize PKU treatment, we thus need to take into account the heterogeneity of the disease, striving toward personalized medicine, i.e., adjusting strictness of treatment to the individual—and, perhaps, age-related—brain vulnerability to high plasma Phe concentrations of a patient. To this purpose, the “unusual” late-diagnosed PKU patients described here may be really helpful.

These “unusual” PKU patients, representing one end of the phenotypic spectrum with regard to neurocognitive outcome in relation to plasma Phe levels, can contribute to our understanding of the relationship between plasma Phe concentrations and brain dysfunction. While the cases described in the present study were selected based on the presence of a relatively normal IQ, many still showed other neurological or psychiatric symptoms. This could be explained by either one or the combination of the following two hypotheses: (1) a generally lower brain vulnerability to high plasma Phe concentrations (possibly due to abnormal transport of Phe from blood to brain or into the neuronal cells) that would prevent (severe) intellectual disability but would still be accompanied by milder cerebral symptoms; or (2) an escape mechanism in one of the metabolic/molecular pathways in the brain, mediating the cerebral responses to high brain Phe concentrations that would prevent certain PKU symptoms (such as intellectual disability), while other symptoms mediated by different pathophysiological mechanisms would still occur. The first hypothesis is supported by the fact that, in some late-diagnosed PKU patients with an unexpected favorable outcome, magnetic resonance spectroscopy (MRS) assessments have shown relatively low brain Phe despite high plasma Phe levels [7,8]. Also, in early treated PKU patients, characteristics of brain Phe uptake were shown to correlate with abnormalities of cerebral white matter as assessed by MRI, as well as IQ [9,10]. However, in 13 PKU patients with reduced brain Phe influx, no DNA polymorphisms were found in the gene encoding for *LAT1*, the predominant transporter for Phe and other large neutral amino acids (LNAA) at the blood–brain barrier (BBB) [11]. Moreover, Phe concentrations in CSF of the above-mentioned reported Case 5 were correspondingly high with the high plasma Phe concentrations. In relation to the second hypothesis, it is of interest that many of the cases presented in this paper showed neurological or psychiatric symptoms that seemed to be different from signs and symptoms seen in classical untreated PKU. Remarkably, whereas seizures are one of the core symptoms of untreated PKU, approximately half of the cases did not have a history of seizures nor EEG abnormalities, so, along with IQ, electrophysiological brain activity seemed to be only relatively mildly affected in these “unusual” PKU patients. It would be of interest to see whether these patients reveal comparable gray matter abnormalities as seen in early treated PKU patients on MRI, as reported by Christ et al. [12]. In contrast, neurological symptoms that were observed more frequently in the “unusual” PKU patients included not only tremors, which can be found in early treated PKU patients, as well, but also headaches, which are not one of the typically reported PKU symptoms. Moreover, many showed one or more psychological, mood, behavioral, and/or social-skill problems. In some cases, these problems were even the primary complaint of the patient and the reason to seek medical consultation that ultimately led to the diagnosis of PKU [13], acknowledging also the possibility that these presenting symptoms are not necessarily due to PKU itself and might also be a non-PKU-related comorbidity. Thereby, the mechanism underlying the difference in brain vulnerability to high plasma Phe concentrations, as well as the question whether this is due to some genetic factor (e.g., a “protective gene” not directly associated with the *PAH* genotype), still remains to be elucidated.

Such differences in cerebral problems resulting from high plasma Phe concentrations is not only observed between individual PKU patients, but also in relation to age, as the clinical picture of suboptimally treated PKU seems to evolve with aging. While high plasma Phe concentrations during childhood are known to primarily affect intellectual functioning, increased plasma Phe concentrations during adolescence seem to result in behavioral issues and executive dysfunction, and during adulthood, they seem to be primarily associated with neurological, psychological, mood, and behavioral problems, as well as deficits in social skills [14,15,16]. In addition, several cases reported here of neurological and/or psychosocial problems that only started in adulthood and thereby led to the late diagnosis of PKU suggest that the adult or aging brain may be more vulnerable to some of these particular pathophysiological mechanisms. While the “unusual” PKU patients somehow seem to be protected against the development of intellectual disability during childhood, these patients may not be protected against other PKU symptoms, including in adulthood.

To conclude, considering the limitations of this study—including a series of case studies with often incomplete clinical documentation and different treatment approaches throughout life—the 16 cases of late-diagnosed and late-treated PKU patients with unexpected favorable intellectual outcomes despite high plasma Phe concentrations, as presented in this study, provide further insight into the inter-individual differences in brain dysfunction of PKU patients. While intellectual functioning in these patients was relatively unaffected, remarkably, they often did present other neurological, psychological, and behavioral problems. Thereby, these “unusual” PKU patients show that the classical symptomatology of untreated or late-treated PKU may have to be rewritten, not only in the sense that intellectual dysfunction is not obligatory, but also in the sense that intellectual functioning does not (re)present the full picture of brain damage due to high plasma Phe concentrations. Furthermore, this may suggest that different mechanisms are involved in the pathophysiology underlying the full clinical entity of brain dysfunction in PKU patients, of which the relative importance seems to differ between patients and possibly also with increased age. In a way, these “unusual” PKU patients are similar to the treated PKU adults who do not really encounter neurocognitive problems, but rather show mood, behavioral, and social-skill issues [14,16]. Further research should aim to better distinguish between PKU patients with respect to their cerebral reactions to high plasma Phe concentrations, including in relation to aging. Investigations to this purpose are underway on both a genetic and functional level, using material of some of the “unusual” PKU patients mentioned in this paper, but also still welcoming material of additional “unusual” PKU patients.

## Figures and Tables

**Table 1:** Cases of late-diagnosed PKU patients who have escaped from intellectual disability despite high plasma Phe concentrations.

Case	Age at Diagnosis (M/F)	Phe (µmol/L)	*PAH* Mutation	IQ	Neurological		Psychological/Psychiatric/Social		Other Investigations
					Normal Findings	Abnormal Findings	Normal Findings	Abnormal Findings	
1A	8 years and 10 months (F)	1800	only tested in brother	102/105	Neurological examination showed no abnormalities	Slow at school and has difficulties focusing			
2	9 years (F)	2400	unknown	(low-) normal	No history of seizures. Finished high school	Intermittent headaches and astigmatism from the age of 44	Poor focus, anger outbursts, anxiety, depression, panic attacks, and episodes of screaming and disorientation at night from 44 years of age	Unable to hold jobs due to behavioural issues	
3A	10 years (F)	>1200	c.1222C > T/p.R408W and c.782G > A/p.R261Q	not tested	Linguistic higher-education degree	Mildly delayed psychomotor development during early childhood			
4	27 years (F)	1500	c.311C > A/p.A104D and c.1315+1G > A/p.L249F	(low-) normal	Completed high school.	Intellectual disability, Parkinson’s features, rigidity, and tremors during age range of 50s–60s	Enrolled nurse	Developed depression during age range of 50s–60s	MRI brain in 60s: mild generalized cerebral atrophy and moderate T2 white matter hyperintensities
5	23 years (F)	1293	c.1222C > T/p.R408W and c.143T > C/p.L48S	81	Completed general school till high school (9 classes)	Headaches, intracranial hypertensive syndrome, learning troubles, and polyneuropathy	Employed	Distrustful on the motives of others, poorly controlled emotions and increased verbal aggression	EEG: irritative changes with residual encephalopathy CT brain: no abnormalities
6	26 years (F)	2580	c.143T > C/p.L48S and c.781C > T/p.R261 *	not tested	High school graduate. No history of seizures. No abnormalities on neurological examination	Worsening headache, scintillating scotoma, photophobia, and phonophobia	No behavioural or psychiatric symptoms		MRI brain: leukoencephalopathy with bilateral white matter hyperintensities
7	52 years (F)	1700	c.194T > C/p.I65T and c.311C > A/p.A104D	not tested		Headache and increasing difficulty with practical tasks from 41 years of age. Short episodes with confusion/loss of memory and involuntary contractions in facial muscles	Assistant nurse	Depression, anxiety, tiredness, and lack of energy from 41 years of age	MRI brain: extensive white matter changes with multiple micro hemorrhages EEG: possible epileptogenic activity
8	29 years (M)	1400	c.168G > T/p.E56D and c.782G > A/p.R261Q	82	No history of seizures	Tremor and slight gait problems from adolescence	Obtained bachelor in social science	Social phobia. Dyslexia. Nonverbal learning difficulties, slow processing speed performance, and strong verbal and reasoning abilities	MRI brain: extensive signal changes in white matter EEG: slightly pathologic frontotemporal peak potentials without epileptogenic activity

PKU: phenylketonuria; M/F: male/female; *PAH*: phenylalanine hydroxylase; IQ: intelligence quotient; MRI: magnetic resonance imaging; EEG: electroencephalography; CT: computed tomography scan.

## Supplementary Materials

The following are available online at https://www.mdpi.com/2072-6643/11/11/2572/s1.

## References

[1] Van Spronsen F.J., van Wegberg A.M., Ahring K., Belanger-Quintana A., Blau N., Bosch A.M., Burlina A., Campistol J., Feillet F., Gizewska M. (2017). Key European Guidelines for the Diagnosis and Management of Patients with Phenylketonuria. Lancet Diabetes Endocrinol..

[2] Van Wegberg A.M.J., MacDonald A., Ahring K., Belanger-Quintana A., Blau N., Bosch A.M., Burlina A., Campistol J., Feillet F., Gizewska M. (2017). The Complete European Guidelines on Phenylketonuria: Diagnosis and Treatment. Orphanet J. Rare Dis..

[3] Vockley J., Andersson H.C., Antshel K.M., Braverman N.E., Burton B.K., Frazier D.M., Mitchell J., Smith W.E., Thompson B.H., Berry S.A. (2014). Phenylalanine Hydroxylase Deficiency: Diagnosis and Management Guideline. Genet. Med..

[4] Van Vliet D., van Wegberg A.M.J., Ahring K., Bik-Multanowski M., Blau N., Bulut F.D., Casas K., Didycz B., Djordjevic M., Federico A. (2018). Can Untreated PKU Patients Escape from Intellectual Disability? A Systematic Review. Orphanet J. Rare Dis..

[5] Blau N., van Spronsen F.J., Levy H.L. (2010). Phenylketonuria. Lancet.

[6] Knox W.E. (1960). An Evaluation of the Treatment of Phenylketonuria with Diets Low in Phenylalanine. Pediatrics.

[7] Weglage J., Moller H.E., Wiedermann D., Cipcic-Schmidt S., Zschocke J., Ullrich K. (1998). In Vivo NMR Spectroscopy in Patients with Phenylketonuria: Clinical Significance of Interindividual Differences in Brain Phenylalanine Concentrations. J. Inherit. Metab. Dis..

[8] Moller H.E., Weglage J., Wiedermann D., Ullrich K. (1998). Blood-Brain Barrier Phenylalanine Transport and Individual Vulnerability in Phenylketonuria. J. Cereb. Blood Flow Metab..

[9] Weglage J., Wiedermann D., Denecke J., Feldmann R., Koch H.G., Ullrich K., Harms E., Moller H.E. (2001). Individual Blood-Brain Barrier Phenylalanine Transport Determines Clinical Outcome in Phenylketonuria. Ann. Neurol..

[10] Weglage J., Wiedermann D., Denecke J., Feldmann R., Koch H.G., Ullrich K., Moller H.E. (2002). Individual Blood-Brain Barrier Phenylalanine Transport in Siblings with Classical Phenylketonuria. J. Inherit. Metab. Dis..

[11] Moller L.B., Paulsen M., Koch R., Moats R., Guldberg P., Guttler F. (2005). Inter-Individual Variation in Brain Phenylalanine Concentration in Patients with PKU is Not Caused by Genetic Variation in the 4F2hc/LAT1 Complex. Mol. Genet. Metab..

[12] Christ S.E., Price M.H., Bodner K.E., Saville C., Moffitt A.J., Peck D. (2016). Morphometric Analysis of Gray Matter Integrity in Individuals with Early-Treated Phenylketonuria. Mol. Genet. Metab..

[13] Allen R.J., Gibson R.M. (1961). Phenylketonuria with Normal Intelligence. Am. J. Dis. Child..

[14] Jahja R., van Spronsen F.J., de Sonneville L.M., van der Meere J.J., Bosch A.M., Hollak C.E., Rubio-Gozalbo M.E., Brouwers M.C., Hofstede F.C., de Vries M.C. (2016). Social-Cognitive Functioning and Social Skills in Patients with Early Treated Phenylketonuria: A PKU-COBESO Study. J. Inherit. Metab. Dis..

[15] Weglage J., Fromm J., van Teeffelen-Heithoff A., Moller H.E., Koletzko B., Marquardt T., Rutsch F., Feldmann R. (2013). Neurocognitive Functioning in Adults with Phenylketonuria: Results of a Long Term Study. Mol. Genet. Metab..

[16] Jahja R., Huijbregts S.C.J., de Sonneville L.M.J., van der Meere J.J., Legemaat A.M., Bosch A.M., Hollak C.E.M., Rubio-Gozalbo M.E., Brouwers M.C.G.J., Hofstede F.C. (2017). Cognitive Profile and Mental Health in Adult Phenylketonuria: A PKU-COBESO Study. Neuropsychology.

