# Supplementary material for: Untreated PKU Patients without Intellectual Disability: What Do They Teach Us?
Source: Nutrients. 2019 Oct 25;11(11):2572. doi: 10.3390/nu11112572 (PMC6893397; doi:10.3390/nu11112572)
Supplement: Supplementary file 1 [file nutrients-11-02572-s001.pdf]

SUPPLEMENTAL MATERIAL

**Detected through affected sibling**

*Supplemental Case 1* is a girl from Egypt who presented at the age of 11y10m with difficulties at school and a sibling who had been diagnosed with PKU some years earlier by newborn screening. She was shown to have PKU as well with a plasma Phe concentration of 1,227 µmol/l, which was confirmed by genetic analysis with a compound heterozygosity for the *PAH* variants c.782G>A/p.R261Q and c.967_969del/p.T323del. She had no history of seizures. Neurocognitive assessment showed a full-scale IQ of 79 (verbal IQ: 86; performal IQ: 74), while neurological assessment did not show any abnormalities.

**Detected through affected/PKU child**

*Supplemental Case 2* is a Danish woman of normal intelligence and without a history of seizures, who has completed one year of business school. At the age of 20 years, she gave birth to a child with microcephaly, and was then found to have a plasma Phe concentration of 980 µmol/l herself with subsequent measurements up to 1,550 µmol/l. Genetic analysis showed compound heterozygosity for the *PAH* variants c.1222C>T/p.R408W and c.1315+1G>A/p.IVS12+1G>A. She started a Phe-restricted diet at the time of her second pregnancy, but this was discontinued and changed for large neutral amino acid treatment after four years. At later age, neuropsychological assessment by the WAIS showed an IQ within the normal range.

*Cases 3 and 4* are two unrelated women from Serbia with comparable stories. At the age of 18y and 21y, they gave birth to a child who was diagnosed with PKU in newborn screening (case 3), and a child with signs of the maternal PKU syndrome (case 4), and these mothers were subsequently found to have PKU with plasma Phe concentrations of approximately

1,200 µmol/l. PKU diagnosis in both patients was further confirmed by genetic analyses showing homozygosity for the *PAH* c.143T>C/p.L48S variant. Neurocognitive examination by the Witti test at the moment of diagnosis showed a normal IQ of 80-85 in both patients. Treatment has never been applied.

*Supplemental Case 5* is a German woman with normal intelligence and without a history of seizures. At age 22 years, she gave birth to a child presenting with features of the maternal PKU syndrome. She was then found to have a plasma Phe concentration of 1,230 µmol/l and promptly started on dietary treatment. At the age of 43 years, neuropsychological assessment by a WAIS-IV showed a disharmonic profile (verbal comprehension: 65; perceptual reasoning: 94; working memory: 89; and processing speed: 83).

**Mental/neurological deterioration at later age**

*Supplemental Case 6* is a male from the USA who had completed 2 years of vocational college and had been working as a plumber and a warehouse keeper. Since his 40s, he had suffered from generalized tonic-clonic and partial complex seizures. At age 48, he began to have episodes of confusion, speech problems, a change in gait, swallowing difficulties, dizziness, tinnitus, and memory loss. MRI performed at 50 years of age showed minor microvascular changes, which increased over the years. At age 57 years, he presented with an acute change in mental status, which was responsive to steroid therapy, and a picture of leukoencephalopathy on MRI. Further investigations because of his history of seizures and a sibling, who had been diagnosed with PKU at the age of 14 years, showed a plasma Phe concentration of 1,080 µmol/l. DNA sequencing revealed a compound heterozygosity for the c.1222C>T/p.R408W and c.745C>T/p.L249F *PAH* variants. Dietary treatment had never been applied. Neurological examination at 57 years of age showed a well oriented but anxious

person, who sometimes forgets directions or appointments and has some difficulty in word finding. Physical examination showed a pupil asymmetry, mild postural and kinetic tremor, and spastic paraplegia.

*Supplemental Cases 7A (male) and B (female)* are two siblings originating from Sudan who presented at the age of 18 years when arriving in the UK with some neurocognitive impairment and a family history of a cousin diagnosed with PKU. There was no history of seizures. They were shown to have plasma Phe concentrations of 1,600 and 1,500 µmol/l, respectively, and dietary treatment in both was promptly initiated. Both cases have attended a special needs school with a life skills course, are independent for activities of daily living, and are employable doing simple jobs such as stacking shelves. The female sibling is comparatively higher functioning; she comes into clinic by herself, has an understanding of the diet and can convey her medical concerns. In addition, both have learned to speak English besides Arabic, since coming to the UK.

*Supplemental Cases 8A and B* are male twins originating from Israel, who both had shown an unremarkable neonatal screening result. Case 8A presented at the age of 3y9m with a developmental delay and seizure disorder and concomitant Tourette's syndrome and attention deficit hyperactivity disorder. Biochemical investigation then showed plasma Phe concentrations up to 1,440 µmol/l and cerebral MRI showed mild abnormalities. Following the diagnosis of PKU, a Phe-restricted diet was instituted on which seizures disappeared. In addition, his twin brother with developmental delay and behavioral-emotional difficulties without additional psychiatric abnormalities nor seizures was investigated and found to have plasma Phe concentrations up to 1,400 µmol/l as well. Similar to his brother, a Phe-restricted

diet was initiated immediately. At age 9 and 12 years, both brothers showed intellectual functioning within the normal range.
